# Effect of low-temperature stress on sperm DNA methylation and oxidative damage during cryopreservation of Tibetan pig semen

**DOI:** 10.14202/vetworld.2026.1747-1758

**Published:** 2026-04-29

**Authors:** Mengqi Duan, Licuo Ze, Yushi Wang, Mingbang Wei, Hongliang Zhang, Peng Shang

**Affiliations:** 1 College of Animal Science, Xizang Agriculture and Animal Husbandry University, Linzhi 860000, Xizang, China; 2 Key Laboratory of Tibetan Pig Genetic Improvement and Reproduction Engineering, Linzhi 860000, Xizang, China; 3 Tibetan Pig Science and Technology Courtyard in Nyingchi, Linzhi 860000, Xizang, China

**Keywords:** cryopreservation, DNA methylation, epigenetics, genome-wide analysis, oxidative stress, sperm quality, Tibetan pig, *in vitro* fertilization

## Abstract

**Background and Aim:**

Cryopreservation of boar semen is widely used for genetic conservation and artificial insemination; however, it induces oxidative stress and molecular alterations that compromise sperm function. Epigenetic modifications, particularly DNA methylation, play a critical role in sperm integrity and fertilization capacity. This study aimed to investigate genome-wide DNA methylation changes and their association with oxidative stress during cryopreservation of Tibetan pig semen.

**Materials and Methods:**

Semen samples were collected from healthy Tibetan pigs and cryopreserved using a standard freeze–thaw protocol. Sperm quality parameters, including motility, viability, and membrane integrity, were assessed post-thaw. Oxidative stress markers, including reactive oxygen species (ROS) levels and antioxidant enzyme activities, were evaluated. Genome-wide DNA methylation profiling was performed using methylated DNA immunoprecipitation sequencing (MeDIP-Seq). Differentially methylated regions (DMRs) were identified, and functional enrichment analyses, including Gene Ontology and Kyoto Encyclopedia of Genes and Genomes pathways, were conducted.

**Results:**

Cryopreservation significantly reduced sperm motility, viability, and membrane integrity, accompanied by elevated ROS levels and disrupted antioxidant defense systems. MeDIP-Seq analysis revealed widespread alterations in DNA methylation patterns between fresh and cryopreserved sperm, with numerous DMRs identified across the genome. These methylation changes were predominantly associated with genes involved in oxidative stress response, apoptosis, energy metabolism, and signal transduction pathways. Functional enrichment analysis highlighted significant involvement of pathways related to mitochondrial function, reactive oxygen species metabolism, and cellular stress responses, indicating a strong link between epigenetic modifications and cryoinjury mechanisms.

**Conclusion:**

Cryopreservation induces substantial oxidative stress and genome-wide alterations in DNA methylation in Tibetan pig sperm. The identified epigenetic changes are closely associated with pathways regulating oxidative stress and cellular integrity, suggesting that DNA methylation plays a crucial role in sperm cryodamage. These findings provide novel insights into the molecular mechanisms underlying cryopreservation-induced sperm dysfunction and may contribute to the development of improved cryopreservation strategies for enhanced reproductive performance.

## INTRODUCTION

As a core epigenetic mechanism, DNA methylation plays a central role in gene expression and genomic stability [1–3]. During spermatogenesis, changes in DNA methylation significantly affect sperm quality, function, and fertilization capacity. Previous studies have demonstrated that DNA methylation not only ensures normal reproduction by maintaining the silencing of transposons before meiosis but is also closely associated with fertilization potential [4–7]. Abnormal methylation may lead to spermatogenic disorders and reduced reproductive performance. Therefore, understanding alterations in sperm DNA methylation is of great significance for improving reproductive outcomes.

The Tibetan pig, a breed endemic to the high-altitude and cold regions of China, is characterized by strong cold tolerance and superior meat quality, making it a valuable local genetic resource. However, due to inconsistent documentation of genetic resources and an incomplete conservation system, cryopreservation technology is particularly important for preserving and utilizing Tibetan pig genetic resources [8, 9]. Semen cryopreservation enables long-term storage of genetic material from superior boars; however, several challenges remain. Pig sperm is highly sensitive to temperature fluctuations, and environmental temperature changes exceeding 2°C can cause excessive loss of membrane lipids and induce cold shock responses, resulting in decreased motility and cellular damage [10].

During freezing, there is increased production of reactive oxygen species (ROS) and reactive nitrogen species (RNS). These molecules attack unsaturated fatty acids in cell membranes, trigger lipid peroxidation, and damage membrane integrity and DNA, ultimately affecting semen quality and fertility potential [11, 12]. Oxidative stress is a key factor contributing to the decline in post-thaw sperm quality [13]. In sperm cells, mitochondria regulate ROS levels, which are essential for capacitation and fertilization under physiological conditions but become detrimental when antioxidant defenses are overwhelmed [14].

Despite extensive research on semen cryopreservation and oxidative stress in pigs, current knowledge remains largely limited to physiological and biochemical aspects, such as membrane integrity, motility, and antioxidant capacity. Although oxidative stress has been well established as a major factor contributing to sperm damage during the freeze–thaw process, the underlying epigenetic mechanisms, particularly those involving DNA methylation, are still poorly understood. Previous studies have highlighted the importance of DNA methylation in regulating gene expression and maintaining sperm function; however, there is a lack of comprehensive genome-wide investigations examining how cryopreservation-induced stress alters methylation patterns in pig sperm.

Furthermore, most existing studies have focused on commercial pig breeds, with minimal attention given to indigenous breeds such as the Tibetan pig, which possess unique adaptive traits to extreme environments. The potential influence of these adaptations on epigenetic stability during cryopreservation has not been explored. In addition, the interaction between oxidative stress and changes in DNA methylation, and how this relationship contributes to cryoinjury and reduced fertility, remains unclear. Importantly, there is also a lack of integrative analyses linking differentially methylated regions with functional pathways and key regulatory genes associated with sperm survival and stress response. Therefore, a systematic investigation combining methylation profiling with functional enrichment analysis is needed to elucidate the molecular basis of cryodamage in Tibetan pig sperm.

The present study aimed to investigate genome-wide DNA methylation changes in Tibetan pig sperm during the freeze–thaw process and to explore their association with oxidative stress–induced cryoinjury. Specifically, this study sought to identify differentially methylated regions across fresh, protectant-treated, and frozen semen samples, and to characterize their genomic distribution and functional significance. In addition, the study aimed to perform Gene Ontology (GO) and Kyoto Encyclopedia of Genes and Genomes (KEGG) pathway enrichment analyses to identify key biological processes and signaling pathways involved in sperm damage and recovery.

Furthermore, this study intended to construct protein–protein interaction networks to identify core regulatory genes associated with cryostress responses. By integrating epigenetic and functional analyses, this research aimed to provide novel insights into the molecular mechanisms underlying sperm cryoinjury and to establish a theoretical foundation for improving semen cryopreservation strategies in Tibetan pigs. Ultimately, the findings are expected to contribute to the conservation of valuable genetic resources and to enhance reproductive efficiency in livestock production systems.

## MATERIALS AND METHODS

### Ethical approval

All procedures involving animals in this study were conducted in strict accordance with institutional, national, and international guidelines for the care and use of animals in research. Ethical approval was obtained from the Animal Ethics Committee of Tibet Agricultural and Animal Husbandry College, China (approval number: XZA-2025-014). The study design and experimental procedures complied with the principles outlined in the Guide for the Care and Use of Laboratory Animals and were consistent with the ARRIVE 2.0 guidelines for reporting animal research.

The animals used in this study were clinically healthy Tibetan boars maintained under standard husbandry conditions, with free access to feed and water. Semen collection was performed using the hand-held method, a non-invasive, routine procedure in livestock management that does not cause pain, injury, or distress to the animals. All efforts were made to minimize animal handling time and to reduce potential stress during sample collection.

No experimental treatments, surgical interventions, or harmful procedures were applied to live animals during the course of this study. All biological samples were collected as part of routine reproductive management practices, and no animals were euthanized specifically for this research.

Laboratory procedures involving semen processing (Beijing Tianyuan Aori Biotechnology Co., Beijing, China), DNA extraction (Tiangen, Beijing, China), and sequencing (Illumina, San Diego, USA) were conducted in accordance with standard biosafety protocols to ensure operator safety and sample integrity. The study adhered to a One Health approach by ensuring ethical responsibility, animal welfare, and scientific rigor in the generation and application of research findings.

### Study period and location

This study was conducted from March 2024 to January 2025. Sample collection was completed at the Tibetan pig breeding base in Zengba Village, Baiba Town, Nyingchi City, Xizang, with an altitude of 3000 m, and all laboratory analyses were carried out at the Key Laboratory of Animal Genetics and Breeding, College of Animal Science, Xizang Agriculture and Animal Husbandry University.

### Animals

Six healthy Tibetan boars aged 6–12 months, with normal reproductive characteristics, were selected as experimental animals.

### Semen collection and initial evaluation

Tibetan boar semen was collected using the hand-held method. Before collection, the relevant areas were cleaned and disinfected, and semen was collected into a sterile collection cup preheated to 37°C to avoid contamination. Immediately after collection, semen samples were evaluated for color, volume, and contamination. Only milky white, odorless, and uncontaminated semen samples were selected. Qualified samples were temporarily stored in a 37°C water bath and subjected to subsequent procedures after preliminary evaluation.

### Semen cryopreservation and protectant treatments

Three groups of semen were established: fresh, protectant-added, and frozen. Freeze protectants I and II were added to fresh semen to form the protectant-added group. The frozen semen group was prepared based on the protectant-added group. Freezing and thawing procedures were performed according to the methods described by Oldenhof *et al*. [15] and Aurich *et al*. [16]. All reagents were supplied by Beijing Tianyuan Aori Biotechnology Co., Ltd. (Beijing, China).

### Sperm motility and quality assessment

Sperm motility was quantitatively assessed using computer-assisted sperm analysis (CASA) software (Beijing Tianyuan Aori Biotechnology Co., Ltd). One to two drops of semen were placed on a pre-heated slide and maintained on a 37°C constant-temperature platform. The CASA system analyzed sperm quality by randomly selecting 8 fields of view per sample, ensuring that at least 1,000 sperm were evaluated.

Microscope parameters were set using a phase-contrast inverted biological microscope (Beijing Tianyuan Aori Biotechnology Co., Ltd.) equipped with a 10× objective lens, and thresholds for identifying sperm motion trajectories were defined according to standard criteria (VCL ≥ 50 μm/s, STR ≥ 80%). Data were collated in Microsoft Excel and analyzed in SPSS version 18.0 (IBM Corp., Armonk, NY, USA) and GraphPad Prism 10 (GraphPad Software, San Diego, CA, USA). Data are presented as mean ± standard deviation. Differences between groups were compared using Student’s t-test. A p-value < 0.05 was considered statistically significant (*p* < 0.05; *p < 0.01).

### DNA extraction and MeDIP-Seq library construction

Genomic DNA was extracted from fresh sperm, protectant-added sperm, and frozen sperm groups using a semen/sperm DNA purification kit (Hangzhou Xinjing Biological Reagent Development Co., Ltd., Hangzhou, China), following the manufacturer’s instructions [16], and stored at −20°C.

Genomic DNA was sheared by sonication into 100–300 bp fragments, followed by 150 bp paired-end sequencing with 10-fold coverage. Fragmented DNA was end-repaired, dA-tailed, and adapter-ligated using the GenSeq® Universal DNA Library Prep Kit (ABclonal, Wuhan, China). Approximately 10% of ligated DNA was reserved as an “Input” control, while the remaining DNA underwent immunoprecipitation using the GenSeq® 5mC MeDIP Kit (ACTIVE MOTIF, Carlsbad, CA, USA).

Both immunoprecipitated and input DNA libraries were amplified using Illumina PCR primers and GenSeq® 2× HiFi PCR Mix (GenSeq Inc., China). Final libraries were quantified using Qubit fluorescence detection (Thermo Fisher Scientific, Waltham, MA, USA), followed by sequencing.

### High-throughput sequencing and quality control

Raw sequencing data were generated using the Illumina HiSeq 4000 platform (Shanghai Yunxu Biotechnology Co., Ltd., Shanghai, China). Following image analysis, base calling, and quality control, initial reads were obtained. Sequencing adapters were removed and low-quality reads were filtered using Cutadapt software (v1.9.1, https://cutadapt.readthedocs.io/), resulting in high-quality clean reads.

### Identification of differentially methylated regions

Clean reads were aligned to the pig reference genome (*Sus scrofa*) using Bowtie2 software (v2.2.4, http://bowtie-bio.sourceforge.net/bowtie2/index.shtml) to generate BAM-format alignment files. Differentially methylated regions were identified using diffReps software (v1.55.4, http://diffreps.readthedocs.io/) with thresholds of p *<* 0.05 and false discovery rate < 0.01.

Methylated sites were subsequently annotated using the UCSC RefSeq database and bedtools (v2.31.0; https://bedtools.readthedocs.io/) and classified into promoter, upstream regulatory, intronic, exonic, and intergenic regions.

### Functional enrichment analysis

Genes associated with differentially methylated regions were annotated using the UCSC RefSeq database (https://genome.ucsc.edu/) and subjected to GO and Kyoto Encyclopedia of Genes and Genomes pathway enrichment analyses to predict biological functions. Enrichment significance was determined using thresholds of p < 0.05 and false discovery rate < 0.01.

### Protein–protein interaction network analysis

The STRING database (https://cn.string-db.org/) was used to construct protein–protein interaction networks of genes associated with the top 20 Kyoto Encyclopedia of Genes and Genomes-enriched pathways. Interaction networks with a medium confidence score (≥0.4) were downloaded and visualized using Cytoscape software (version 9.1.21, https://cytoscape.org/).

Hub genes were identified using the maximal clique centrality algorithm in the Cytoscape CytoHubba plugin (version 1.23, https://apps.cytoscape.org/apps/cytohubba), with the top four nodes selected based on their scores.

## RESULTS

### Semen quality analysis

**Figure 1:**
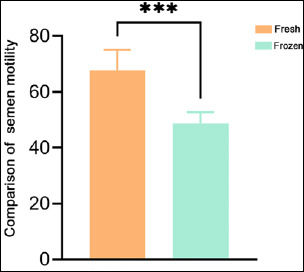
Comparison of sperm viability between fresh and frozen-thawed Tibetan pig semen.

As shown in Table 1, the initial sperm motility of the six Tibetan boars exceeded 50%, meeting the standard required for frozen semen production. Additionally, frozen-thawed sperm exhibited a significant reduction in motility compared to fresh sperm, with a highly significant difference between the two groups (p < 0.001) (Figure 1A). These results indicate that the freeze–thaw process causes irreversible damage to sperm, leading to reduced motility after thawing.

**Table 1:** Results of vitality tests after thawing of raw semen and frozen semen.

Breed	Number	Original sperm motility (%)	Average sperm motility (%)	Frozen-thawed sperm motility (%)	Average sperm motility (%)
Tibetan pig	005	68.1 ± 4.82	67.6 ± 6.83	48.9 ± 5.61	46.9 ± 5.11
Tibetan pig	035	78.5 ± 4.13	—	49.9 ± 5.18	—
Tibetan pig	015	61.6 ± 4.01	—	46.0 ± 6.45	—
Tibetan pig	011	72.8 ± 4.51	—	50.1 ± 4.96	—
Tibetan pig	013	57.7 ± 3.75	—	54.7 ± 4.75	—
Tibetan pig	009	67.4 ± 4.75	—	43.2 ± 3.0	—

Note: Values are expressed as mean ± standard deviation. Statistical significance was determined using a paired t-test. p < 0.05, p < 0.01, p < 0.001*,* ns = no significant difference.

### Sequencing data quality assessment

Sequencing quality control was performed for all groups: fresh sperm, protectant-added sperm, and frozen sperm. The number of raw reads varied among samples; however, Q30 values exceeded 98.3% and clean read rates approached 99.6%, indicating high sequencing quality suitable for further analysis (Table 2).

**Table 2:** Sample quality control data statistics.

Sample	raw_reads	Q30	clean_reads	clean_rate
Fresh1-IP	40519218	98.78%	40461344	99.86%
Fresh2-IP	43933438	98.77%	43838688	99.78%
Fresh3-IP	46183990	98.75%	46111222	99.84%
protectant1-IP	42746272	98.78%	42670238	99.82%
protectant2-IP	42028552	98.73%	41958522	99.83%
protectant3-IP	44689060	98.78%	44618026	99.84%
frozen1-IP	42822012	98.70%	42753138	99.84%
frozen2-IP	37304096	98.64%	37226634	99.79%
frozen3-IP	40170348	98.73%	40106976	99.84%
Fresh-Input	40240506	98.79%	40137784	99.74%
protectant-Input	36291624	98.58%	36169678	99.66%
frozen-Input	41418044	98.37%	41298266	99.71%

### Identification of methylation regions (enrichment peaks)

**Figure 2:**
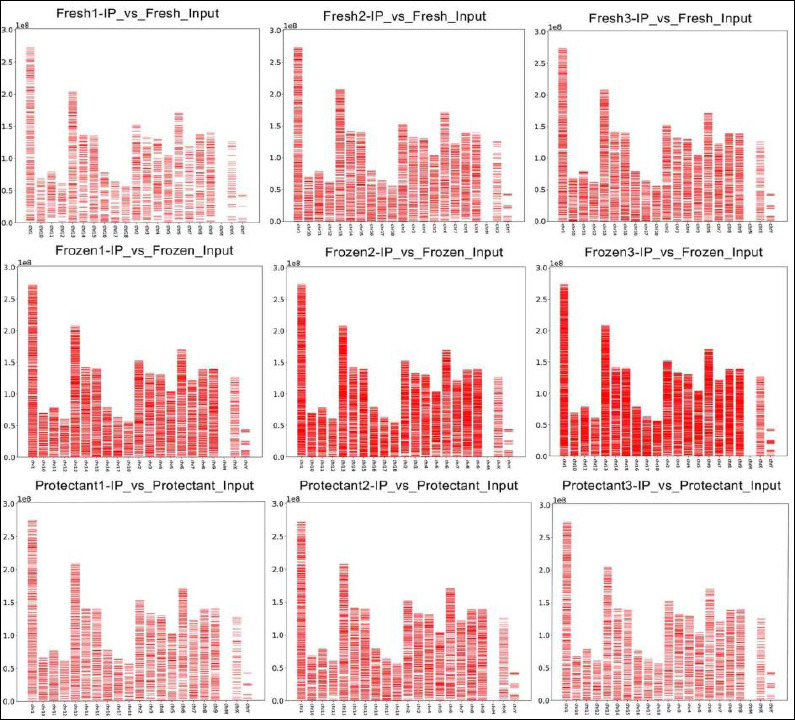
Distribution of methylation regions (enrichment peaks) on chromosomes. The X-axis represents chromosome number, and the Y-axis indicates genomic length in megabases (Mb). The red bands denote methylation regions detected as enrichment peaks, and their positions indicate methylation loci.

A total of 7,991 methylated regions were identified in the fresh group, 13,669 in the frozen group, and 7,920 in the protectant-added group. The chromosomal distribution of methylated regions in each treatment group is presented in Figure 2.

### Methylation region (enrichment peaks) annotation

**Figure 3:**
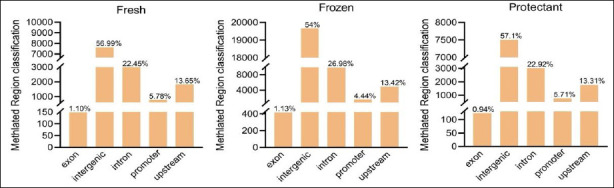
Distribution of methylation regions across various transcription elements. The X-axis displays transcription elements, including exons, intergenic regions, introns, promoters, and upstream regions, and the Y-axis shows the proportion of reads.

Analysis of methylation levels across genomic regions revealed distinct distribution patterns. The highest methylation levels were observed in intergenic regions, whereas exonic regions exhibited the lowest levels across all groups (Figure 3). These findings suggest that intergenic methylation may play a regulatory role in gene expression and genomic stability, whereas low methylation in exons may support normal transcription and translation.

### Identification and annotation of differentially methylated regions

**Figure 4:**
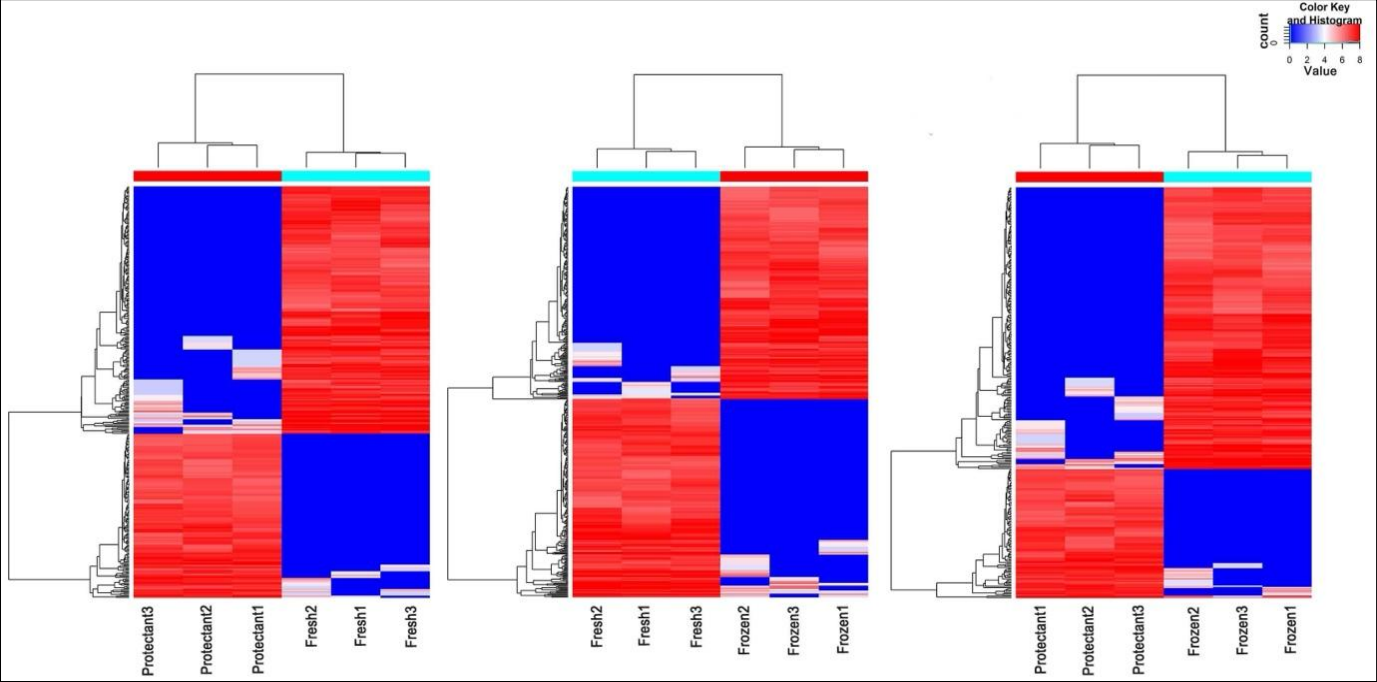
Regional distribution of differential methylation in different groups. Red represents hypermethylated sites and blue represents hypomethylated sites. Each row corresponds to a gene, and each column corresponds to a sample.

**Figure 5:**
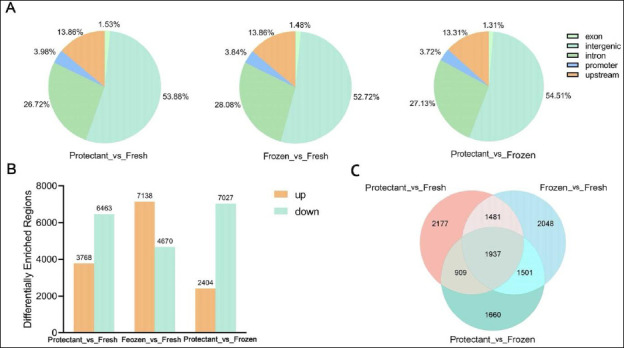
(A) Distribution of differentially methylated regions on different gene functional elements, (B) comparison of upregulation and downregulation among different groups, (C) and number of target genes among different groups.

In this study, diffReps software was used to identify differentially methylated regions. A total of 31,470 differentially methylated regions were identified (Figure 4). Compared with the fresh group, the protectant-added group had 3,768 upregulated and 6,463 downregulated regions, whereas the frozen group had 7,138 upregulated and 4,670 downregulated regions. When comparing the protectant-added group with the frozen group, 2,404 regions were upregulated and 7,027 were downregulated (Figure 5B).

Functional annotation revealed that 53.5% (16,880) of regions were located in intergenic regions, followed by 27.5% (8,609) in intronic regions, 13.7% in upstream regions, 3.8% in promoter regions, and 1.5% in exonic regions (Figure 5A). Further analysis across the three groups identified 1,937 target genes (Figure 5C).

### GO and Kyoto encyclopedia of genes and genomes pathway analysis

**Figure 6:**
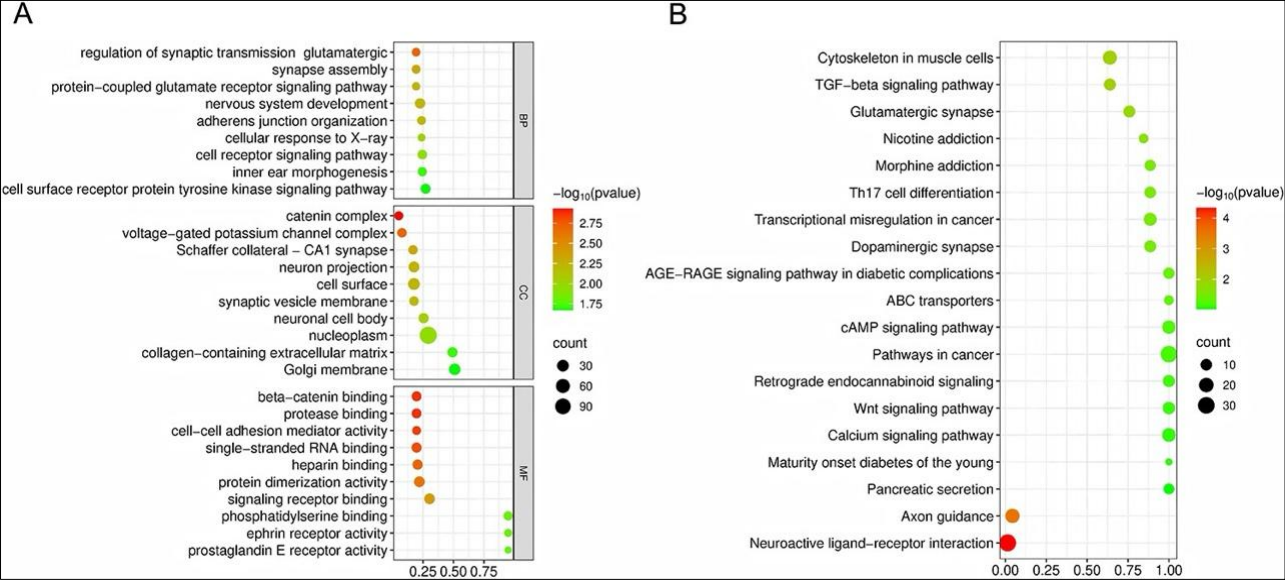
Differentially methylated region enrichment results in Gene Ontology and Kyoto Encyclopedia of Genes and Genomes analyses. (A) Gene Ontology analysis and (B) pathway enrichment analysis. The vertical axis represents pathway names, the horizontal axis represents the number of genes, and the color of the dots corresponds to different p-value ranges, with darker colors indicating smaller p-values.

To explore the biological functions of differentially methylated genes and understand how cold stress affects DNA methylation in Tibetan pig semen, GO annotation analysis was performed. The top 10 enriched terms included genes involved in DNA damage repair, oxidative stress response, cell membrane integrity, cell junctions, motility, and lipid metabolism (Figure 6A).

Kyoto Encyclopedia of Genes and Genomes pathway analysis revealed three oxidative stress-related signaling pathways: transforming growth factor beta signaling, advanced glycation end product–receptor for advanced glycation end product signaling in diabetic complications, and calcium signaling. These pathways are likely tocontribute in damage to sperm plasma membranes and cellular structures during cryopreservation, thereby reducing sperm quality (Figure 6B).

### Protein–protein interaction network analysis

**Figure 7:**
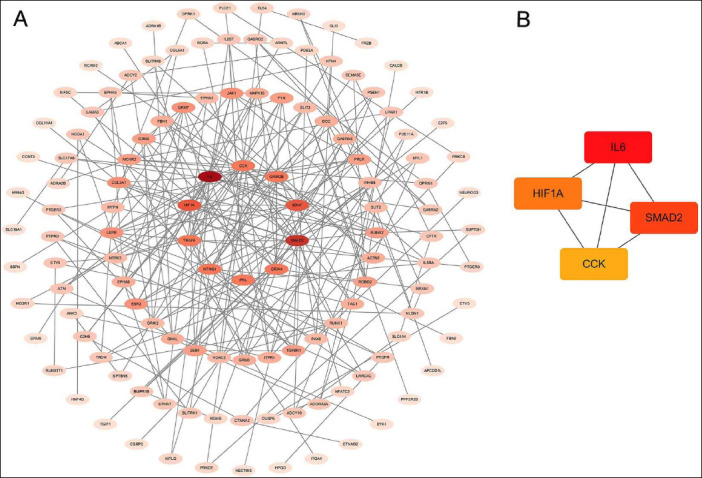
Visualization of the protein–protein interaction network. (A) Protein–protein interaction network diagram of Kyoto Encyclopedia of Genes and Genomes-associated genes and (B) interaction network of hub genes. Each line represents an interaction between proteins encoded by genes. Gene rankings are indicated by different colors, with darker colors indicating more central genes.

To further investigate the molecular mechanisms underlying semen cryoinjury, a protein–protein interaction network was constructed for genes associated with the top 20 Kyoto Encyclopedia of Genes and Genomes-enriched pathways (Figure 7A).

Several core hub genes were identified: *IL6*, *SMAD2*, *HIF1A*, and *CCK* (Figure 7B). Among these, *SMAD2*, a key regulator in the transforming growth factor beta signaling pathway, may influence the sperm cell response to freezing stress when its function is disrupted.

## DISCUSSION

### Oxidative stress and cryoinjury

Since Spallanzani’s initial cryopreservation experiment on mammalian semen in 1766, semen cryopreservation has evolved into a key biotechnological tool for livestock breeding and genetic resource conservation [17]. This technology is particularly critical for the conservation of rare breeds, such as the Tibetan pig. Our study observed a significant reduction in post-thaw sperm viability compared to fresh semen (p < 0.001), consistent with findings by Barbas *et al*. [18]. This decline is attributed to irreversible damage induced by cold stress and oxidative damage during freeze–thaw cycles.

Physiologically, sperm maintain redox homeostasis through endogenous antioxidants and environmental exogenous antioxidants [11, 12]. However, cryopreservation disrupts membrane structures through low temperatures and ice crystal formation, leading to cell death. The accumulation of dead sperm elevates reactive oxygen species levels while reducing antioxidant enzyme activity, thereby exacerbating oxidative stress, impairing semen quality, and diminishing fertilization potential [19, 20].

These observations are consistent with Spinaci *et al*. [21], who reported that freeze–thaw processes compromise plasma membrane and DNA integrity, resulting in reduced sperm motility and highlighting oxidative stress as a primary driver of cryoinjury. Strategies to mitigate these effects, such as supplementation with natural phenolic antioxidants (e.g., phenolic acids, stilbenes, and flavonoids), have shown promise in scavenging reactive oxygen species and protecting sperm [22, 23].

### Epigenetic regulation of sperm viability

In the present study, a total of 31,470 differentially methylated regions were identified using methylated DNA immunoprecipitation sequencing technology, with enrichment predominantly in intergenic (53%) and intronic (27%) regions, consistent with findings reported by Ye et al. [24]. High levels of intergenic and intronic methylation may indirectly regulate gene expression by altering chromatin structure or interacting with transcription factors [25].

Through Kyoto Encyclopedia of Genes and Genomes enrichment analysis, key pathways associated with cryostress responses were identified, including transforming growth factor beta signaling, advanced glycation end product–receptor for advanced glycation end product signaling, and calcium signaling, all of which are closely linked to oxidative stress and sperm function. The transforming growth factor beta signaling pathway regulates the expression of antioxidant enzymes, such as superoxide dismutase and glutathione peroxidase, through SMAD2, thereby mitigating oxidative damage [26, 27].

In the advanced glycation end product–receptor for advanced glycation end product pathway, activation by advanced glycation end products induces overproduction of reactive oxygen species via NADPH oxidase [28, 29], leading to lipid peroxidation, DNA damage, and reduced sperm motility [30, 31]. This mechanism, previously associated with diabetes-related reproductive dysfunction, is now extended to cryostress conditions.

The calcium signaling pathway enhances antioxidant capacity by upregulating superoxide dismutase and glutathione peroxidase [32, 33] and plays a crucial role in sperm capacitation, where calcium influx drives progressive motility and hyperactivation [34].

### Screening and functional analysis of key genes

To further elucidate the molecular mechanisms underlying semen freezing injury, core hub genes were identified from the top 20 enriched pathways: *IL6*, *SMAD2*, *HIF1A*, and *CCK*.

*SMAD2*, as a key regulator of the transforming growth factor beta signaling pathway, plays an important role in spermatogenesis and sperm survival. Its copy number variation has been associated with reproductive performance [35, 36]. *IL6*, a pro-inflammatory cytokine, activates the STAT3 pathway under cryopreservation stress, thereby enhancing inflammatory responses and reactive oxygen species production, which contributes to sperm damage [37–39].

HIF1A is aberrantly activated under oxidative stress conditions, promoting apoptosis and impairing sperm function [40, 41]. *CCK*, a neuropeptide involved in regulating sperm motility, may influence sperm vitality through the calcium signaling pathway [42, 43]. The identification of these key genes provides new insights into the molecular regulation of cryostress; however, the precise mechanisms remain to be investigated.

The present study provides a comprehensive analysis of DNA methylation dynamics in Tibetan pig sperm during the freeze–thaw process and highlights their association with oxidative stress–induced cryoinjury. A significant reduction in post-thaw sperm motility (p < 0.001) was observed, confirming that cryopreservation induces substantial functional damage. Genome-wide methylation profiling identified 31,470 differentially methylated regions, predominantly distributed in intergenic and intronic regions, suggesting their regulatory role in gene expression. Functional enrichment analyses revealed that key pathways, including transforming growth factor beta signaling, advanced glycation end product–receptor for advanced glycation end product signaling, and calcium signaling, are closely associated with oxidative stress responses and sperm function. Furthermore, key hub genes, including *IL6*, *SMAD2*, *HIF1A*, and *CCK*, were identified as potential regulators of cryostress-mediated sperm damage.

From a practical perspective, these findings provide valuable molecular insights for improving semen cryopreservation strategies in Tibetan pigs. Targeted approaches, such as antioxidant supplementation and modulation of epigenetic pathways, may help mitigate oxidative damage and enhance post-thaw sperm quality, thereby improving reproductive efficiency and supporting the conservation of valuable genetic resources.

A major strength of this study is the integration of genome-wide methylation profiling with functional and network analyses, providing a systematic understanding of epigenetic regulation under cryostress conditions. Additionally, the focus on an indigenous breed adapted to extreme environments offers novel insights into breed-specific responses that are often overlooked in conventional studies.

However, several limitations should be acknowledged. The study was conducted on a single breed with a relatively small sample size, and the findings were not validated using gene expression or protein-level analyses. Moreover, causal relationships between methylation changes and functional outcomes were not experimentally confirmed.

Future research should focus on multi-breed validation, functional characterization of identified hub genes, and integration of transcriptomic and proteomic approaches to elucidate regulatory mechanisms. In addition, experimental validation through targeted interventions, such as antioxidant treatments or epigenetic modifiers, will be essential for translating these findings into practical applications.

In conclusion, this study provides the first comprehensive epigenetic insight into sperm cryoinjury in Tibetan pigs and establishes a theoretical foundation for improving cryopreservation efficiency through epigenetic and oxidative stress–targeted strategies, thereby contributing to sustainable livestock production and genetic resource conservation.

## DATA AVAILABILITY

The data that support the findings of this study are available from the corresponding author upon reasonable request.

## AUTHORS’ CONTRIBUTIONS

MD and LZ: Conceptualization, methodology, data curation, and writing–original draft preparation. MD: Resources and writing–review and editing. YW and LC: Validation and formal analysis, and software. MW: Investigation and supervision. PS and HZ: Visualization, project administration, and funding acquisition. All authors have read and approved the final manuscript.
